# ST-Segment Elevation Myocardial Infarction (STEMI) Management in the Era of COVID-19: A Systematic and Critical Review of Published Guidance Reports

**DOI:** 10.31083/j.rcm2402054

**Published:** 2023-02-06

**Authors:** Konstantinos G Kyriakoulis, Ioannis P Trontzas, Ioannis G Kyriakoulis, Dimitrios Terentes-Printzios, Nikolaos-Georgios Papageorgiou, Eleni Fyta, Elias Kotteas, Anastasios Kollias

**Affiliations:** ^1^Third Department of Medicine, School of Medicine, National and Kapodistrian University of Athens, Sotiria Hospital, 11527 Athens, Greece; ^2^1st Department of Cardiology, Athens Medical School, National and Kapodistrian University of Athens, Hippokration Hospital, 11527 Athens, Greece

**Keywords:** consensus, COVID-19, fibrinolysis, guideline, percutaneous coronary intervention, STEMI

## Abstract

**Background:**

Coronavirus disease 2019 (COVID-19) had a major impact on healthcare systems worldwide. During the early phase of the pandemic many elective procedures were postponed. At the same time, the safe and effective management of medical emergencies such as ST-segment elevation myocardial infarction (STEMI) has been a challenge.

**Methods:**

A systematic literature search was conducted aiming to identify published guidance reports by national or international societies regarding the management of patients suffering STEMI in the era of COVID-19.

**Results:**

Among 1681 articles initially retrieved, six fulfilled the inclusion criteria and were included in the systematic review. Two reports were international consensus documents, while four reports were national guidance statements from Asian countries (Taiwan, India, Iran, and China). Most documents were drafted during the early phase of the pandemic. According to the international consensus documents, percutaneous coronary intervention (PCI) should be regarded as the reperfusion method of choice. On the other hand, in three out of four national consensus statements (Taiwan, Iran and China) fibrinolysis was considered as the reperfusion method of choice for STEMI in suspected/confirmed patients with COVID-19, unless contraindicated or in the presence of high cardiovascular risk clinical features. Authors of all documents underlined the need for early COVID-19 testing in patients with STEMI to better determine the next therapeutical steps.

**Conclusions:**

National and international consensus statements for STEMI management in the era of COVID-19 have been published mainly during the early peak phase of the pandemic. Authors recognise that these recommendations are mainly based on expert opinions and observational data. As global immunization rates increase and methods for rapid COVID-19 detection are widely available, the implementation of traditional evidence-based practices used before the pandemic is becoming more feasible.

## 1. Introduction

Since its outbreak, coronavirus disease 2019 (COVID-19) had a major impact on healthcare systems and practices [1]. Resource-saving strategies and patients’/physicians’ concerns on COVID-19 transmission led many appointments for chronic issues and elective procedures to be postponed [1]. Great concerns have been raised for the proper function of the emergency departments, as resources were saved for COVID-19, while at the same time non-COVID-19 related emergencies could not be neglected [2].

Myocardial infarction (MI), in particular ST-segment elevation myocardial infarction (STEMI) admissions for percutaneous coronary intervention (PCI), have declined during the pandemic [3, 4, 5]. This trend though should be interpreted with caution as it cannot be necessarily translated to absolute decrease of STEMI cases, but perhaps implies a delayed or hesitant admission of STEMI patients due to fear of COVID-19 transmission, with a subsequent increase of out-of-hospital cardiac arrest events [6]. Interestingly, STEMI cases presented a significant delay to hospital admission and management (symptom-to-call and call-to-balloon times) as well as higher troponin levels and worse prognosis compared to STEMI cases before the pandemic [7, 8, 9].

The need to balance exposure risks and patients’ benefits has been highlighted by many medical societies (mainly national) which have suggested fibrinolysis to a more prominent point of the STEMI reperfusion management algorithm; however, this strategy was not universally adopted, especially by international medical societies [10, 11]. This study aimed to systematically review the literature to identify published guidance reports by national or international societies on STEMI management in the era of COVID-19.

## 2. Materials and Methods

### 2.1 Search Strategy

A systematic PubMed search was conducted in line with the Preferred Reporting Items for Systematic Reviews and Meta-Analyses (PRISMA) recommendations independently by two investigators (KGK and IGK) [12]. Literature search was conducted using the algorithm *(“coronavirus 2019” OR “2019-nCoV” OR “SARS-CoV-2” OR “COVID-19” OR COVID) AND (STEMI OR “ST-elevation” OR “ST elevation” OR “ST-segment” OR “ST segment” OR “myocardial infarction”)* until August 8, 2022. Articles were also selected from references of relevant articles and by hand search. Disagreements were resolved by consensus with a senior author (AK).

### 2.2 Selection of Studies

Eligible studies were full-text articles in English that included consensus/guidance/guidelines reports or position statements/recommendations by national or international societies on the management of STEMI in the era of COVID-19. Guidelines, recommended management strategies and algorithms derived by single centers, dedicated hospitals or opinions by experts were not deemed to be eligible for inclusion.

### 2.3 Data Extraction

Data concerning the origin of studies and the main recommendations and strategies regarding STEMI management in the era of COVID-19 pandemic were extracted, tabulated and reviewed by all authors.

## 3. Results

**Fig. 1.**
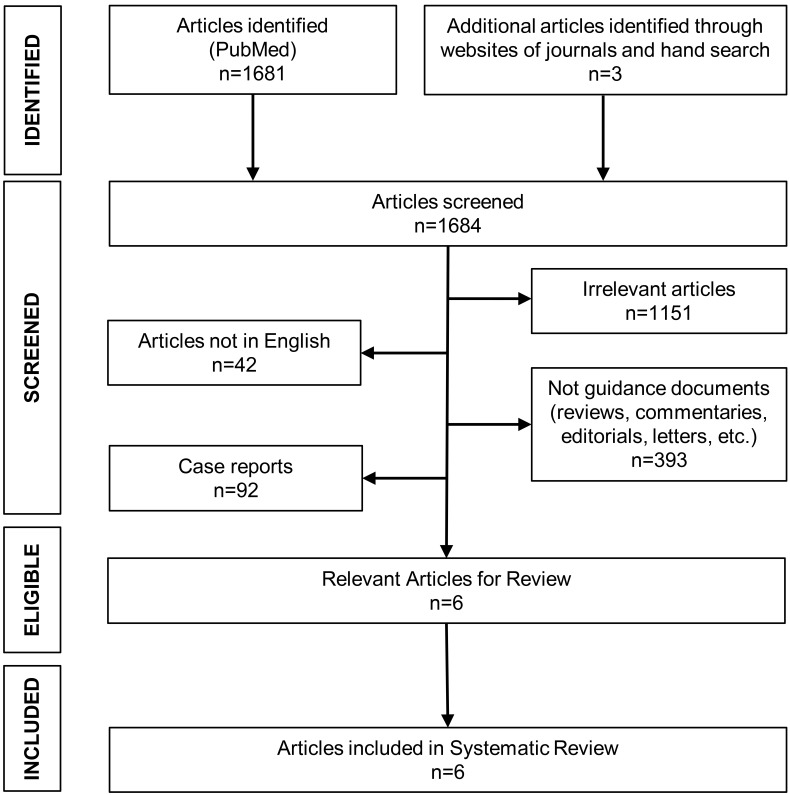
**Flowchart for study selection process of included studies**.

**Fig. 2.**
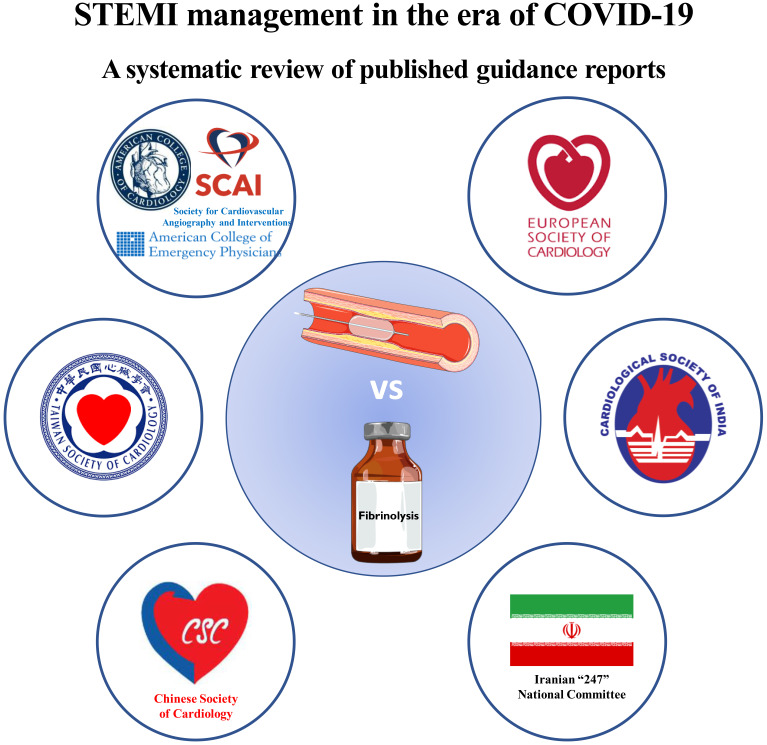
**Visual summary of the present systematic review**.

Among 1681 articles initially retrieved, six fulfilled the inclusion criteria and were included in the systematic review [13, 14, 15, 16, 17, 18]. Search strategy and flowchart for the selection of studies are presented in Fig. 1. Two studies were international consensus documents [13, 14], while the rest four studies were national guidance reports from Asian countries: Taiwan [15], India [16], Iran [17], and China [18]. In Table 1 (Ref. [13, 14, 15, 16, 17, 18]) the main principles of STEMI management according to each society’s guideline document are described. In Fig. 2 a graphical visual summary of the present systematic review is available.

**Table 1.** **Current guidelines for STEMI management in suspected/confirmed COVID-19 patients**.

Society		Indications	
		PCI	Fibrinolysis
International societies	European Society of Cardiology [13]	Within 180 minutes from STEMI diagnosis (preferable time limit of 120 minutes + accepted delay of 60 minutes) and performed in facilities approved for the treatment of COVID-19 patients in a safe manner for healthcare providers and other patients	If primary PCI is not available within 180 minutes from STEMI diagnosis
	Society for Cardiovascular Angiography and Interventions (SCAI), American College of Cardiology (ACC), and the American College of Emergency Physicians (ACEP) [14]	Within 120 minutes from first medical contact in non-PCI capable hospitals and 90 minutes for patients presenting to a primary PCI center	If primary PCI is not available within 120 minutes from first medical contact in non-PCI capable hospitals, consider pre-transfer (to primary PCI-capable hospital) fibrinolysis based on clinical status, transfer delays, team-specific details
National societies	Taiwan Society of Cardiology [15]	If fibrinolysis contraindication or failure	Treatment of choice
	Cardiological Society of India [16]	Treatment of choice (preferred also in case of delayed presentation >12 h and mild/moderate pneumonia)	If timely (<12 from symptom onset) primary PCI is not available. Also preferred in small towns and villages
	Iranian “247” National Committee [17]	Treatment of choice in (i) non-critical illness/pneumonia, (ii) fibrinolysis contraindication or failure, (iii) high risk features present (hemodynamic instability, intractable arrhythmias, anterior or more than one territory MI), (iv) if primary PCI capable center with PPE is available	Treatment of choice in (i) non-critical illness/pneumonia, (ii) if not contraindicated, (iii) high risk features not present (hemodynamic instability, intractable arrhythmias, anterior or more than one territory MI), (iv) if primary PCI capable center with PPE is not available
	Chinese Society of Cardiology [18]	If fibrinolysis contraindication or failure	Treatment of choice within 12 h from symptom onset

COVID-19, coronavirus disease 2019; MI, myocardial infarction; PCI, percutaneous coronary intervention; PPE, personal protective equipment; STEMI, ST-segment elevation myocardial infarction.

### 3.1 International Guidance Documents

#### 3.1.1 European Society of Cardiology (ESC) 

ESC guidance recommendations were primarily accessible through an online website source (currently unavailable) receiving regular updates as evidence was accumulated during the course of the pandemic [19]. A detailed *“Change history log”* was found in this website, describing, and explaining all changes and steps that led to final document since its first draft. In June 2020, the European Association of Percutaneous Cardiovascular Interventions (EAPCI) published a Position Paper regarding the invasive management of acute coronary syndromes in the era of COVID-19 [20]. However, the two most recent ESC consensus documents were published in March and June 2022, respectively. The first document deals with the epidemiology, pathophysiology, and diagnosis of cardiovascular diseases during COVID-19 pandemic [21], whereas the second document deals with care pathways, treatment, and follow-up of patients [13].

In all documents produced by ESC, it is strongly emphasized that all STEMI patients should be initially managed as if they were COVID-19 positive. Subsequently, all patients should be tested for COVID-19 as soon as possible. Reperfusion therapy within 120 minutes from STEMI diagnosis remains the cornerstone for STEMI management. Primary PCI is the preferred reperfusion method, as long as COVID-19 dedicated, and equipped facilities that can assure safety of medical personnel and other patients are available. Further delay of 60 minutes is accepted, given unavoidable delays during the pandemic, e.g., use of personal protective equipment (PPE). If the target of 180 minutes cannot be reached and fibrinolysis is not contraindicated, then fibrinolysis should be the alternative option.

#### 3.1.2 Society for Cardiovascular Angiography and Interventions (SCAI), American College of Cardiology (ACC), and the American College of Emergency Physicians (ACEP)

This consensus statement endorsed by three highly prestigious medical societies was published in 2020 simultaneously in two different recognized medical journals (*Journal of the American College of Cardiology* and *Catheterization and Cardiovascular Interventions*) [14]. Similar to the ESC report, all patients presenting with STEMI should be initially considered as COVID-19 positive. Testing for COVID-19 (ultra-rapid assays if possible) should be conducted as soon as possible. The basic approach of this consensus depends on whether the patient is presented to a primary PCI Center or not. In the first case, primary PCI is the standard of care within 90 minutes of first medical contact, as long as appropriate PPE and resources are available. Otherwise, fibrinolysis should be considered. In case of a patient presenting in a non-primary PCI Center, transfer and primary PCI is the standard of care within 120 minutes of first medical contact, otherwise fibrinolysis should be considered. Transfer to a primary PCI center following initial fibrinolysis has been the standard of care in pre-COVID-19 era and should also be considered during the pandemic [22].

### 3.2 National Guidance Documents

#### 3.2.1 Taiwan Society of Cardiology 

In their consensus report, physicians of the Taiwan Society of Cardiology recognize primary PCI as the major and most important reperfusion strategy for STEMI [15]. However, in the era of COVID-19 and based on preliminary studies derived mainly from China, they do also recognize that optimal door-to-device time cannot be properly achieved for suspected/confirmed COVID-19 patients. Pandemic related delays are inevitably increased and most of the times beyond the acceptable therapeutic time window. As a result, fibrinolysis is the first step of their algorithm for STEMI management. PCI is considered either in case of fibrinolysis contraindications or failure. This document focuses only on suspected or confirmed COVID-19 patients (based on symptoms or history of close contact with confirmed COVID-19 patient) presenting with STEMI. For suspected cases, testing to rule out COVID-19 should be performed as soon as possible.

#### 3.2.2 Cardiological Society of India 

The recommendations of Cardiological Society of India highlight the need to categorize patients presenting with STEMI in 3 groups according to the possibility of suffering COVID-19 [16]. Group A: confirmed cases of COVID-19 with a positive test result, Group B: suspected COVID-19 cases (based on symptoms and history of travel or contact with a definite COVID-19 patient), and Group C: low risk for COVID-19 or COVID-19 negative patients. Due to the shortage of diagnostic tools, Groups A and B should be better grouped as one Group (suspected or confirmed COVID-19 cases) and managed in the same way. For COVID-19 negative or low risk STEMI patients (Group C), treatment following pre-pandemic recommendations is reasonable. For suspected/confirmed COVID-19 cases primary PCI is the preferred method of reperfusion if feasible. PCI is also preferred in case of delayed presentation (>12 h) or in case of fibrinolysis failure. Fibrinolysis is considered as first step if timely primary PCI is not feasible, e.g., in STEMI cases in small towns and villages.

#### 3.2.3 Iranian “247” National Committee

The Iranian “247” National Committee has adopted a more complex algorithm for the management of STEMI in the era of COVID-19 [17]. Considering the low diagnostic value and performance of current diagnostic tools for COVID-19, patients are either definite or indefinite cases, still without reporting certain criteria for this grouping strategy. For all cases, when PCI is preferred, PPE should be applied, given the high transmission rate of the virus, the possibility of transmission by asymptomatic patients and the failure of current diagnostic tools to safely and adequately diagnose or exclude COVID-19 infection. The authors of these guidance report also grouping of patients depending on the hours after chest pain onset upon presentation to emergency department (≤12 h or >12 h). Fibrinolysis is the main reperfusion strategy for patients admitted in less than 12 h after chest pain onset, unless they are critically ill and high-risk features of MI are present. PCI is preferred in non-critically ill patients, if fibrinolysis is contraindicated or failed, when high-risk features of MI are present and when a primary PCI capable center with PPE is available.

#### 3.2.4 Chinese Society of Cardiology

The Chinese Society of Cardiology was the first society to publish consensus guidance report regarding STEMI management (and cardiovascular emergencies in general) during the pandemic [18]. According to the Chinese Society of Cardiology consensus document, a simple algorithm is suggested. Emphasis is initially given in timely testing, aiming to rule-out COVID-19 in suspected/possible cases. Fibrinolysis is the preferred method of reperfusion for suspected/confirmed COVID-19 patients presenting within 12 h from symptoms onset. PCI is indicated only in case of fibrinolysis contraindication or failure.

## 4. Discussion 

The aim of the present systematic review was to systematically review the literature to identify published guidance reports, recommendations, and consensus statements by national or international societies on the management of STEMI patients in the era of COVID-19 pandemic. Six documents were identified and included in the systematic review, among which two were published by international societies [13, 14] and four by the national societies of Taiwan [15], India [16], Iran [17], and China [18].

COVID-19 infection had a major impact on healthcare systems and practices [1, 2]. Measures were globally applied to save resources and minimize infection transmission among patients and healthcare professionals. Most elective procedures were postponed and questions concerning the proper management of acute health urgent and emergent situations, related or not to COVID-19 infection were raised. In this context, new guidance reports sharing current experience and guiding professionals on how to act during the pandemic were necessary. Several hospitals and experts shared their primitive experience and suggested management pathways before national or international documents were available [10, 23, 24, 25, 26]. Moreover, many countries produced their own national documents applied to their health systems. These reports were not drafted in the English language, rendering their universal applicability problematic [27, 28].

**Fig. 3.**
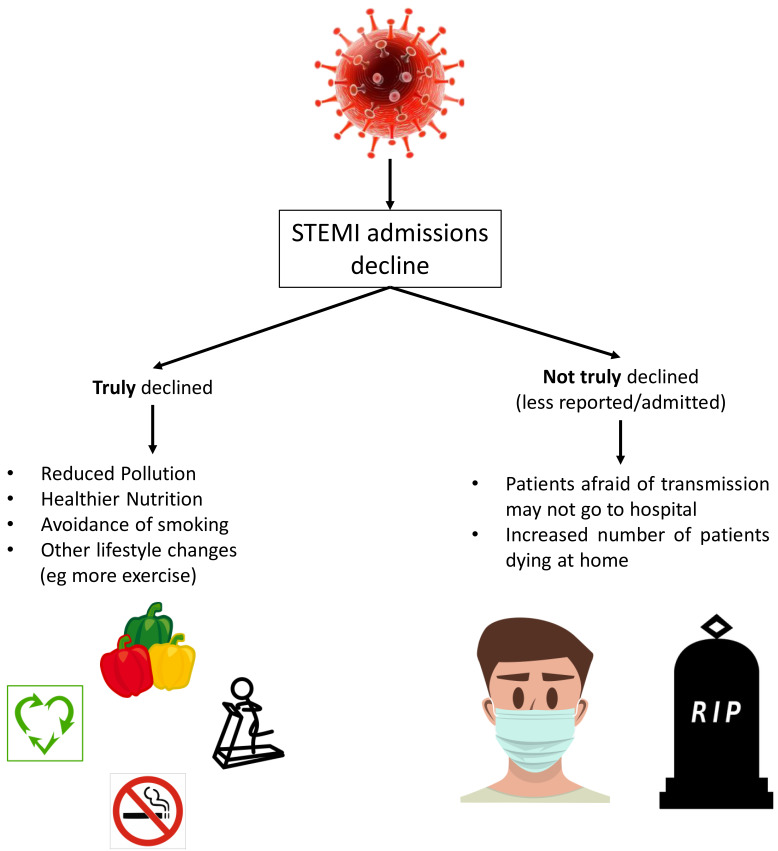
**Possible mechanisms and reasons of STEMI admissions decrease during COVID-19 pandemic**.

Numerous reports commented on STEMI admissions decline during the pandemic, either in COVID-19 positive or negative patients [4, 7, 29, 30, 31]. Whether this is a true decline remains a source of debate. During the pandemic and quarantine period, many factors could attribute to a true decrease of acute events. Less air pollution, better nutritional habits, less smoking or other lifestyle modifications such as more exercise could certainly play an important, beneficial and protective role [29]. However, most researchers agree that STEMI cases decrease (STEMI incidence has declined by 30–70% in the Unites States of America and Italy during the early phase of the pandemic [29]) is mainly attributed to decreased reporting or fewer admissions possibly due to patients’ fear of COVID-19 transmission [29] (Fig. 3). An interesting point to discuss is that although admissions for STEMI seemed to decrease, STEMI cases presented with worse clinical manifestations and outcomes [7, 8, 9, 32]. This could be attributed to delayed patients’ admission (possibly due to the fear of transmission) [7, 8] or to virus-related pathophysiological pathways (inflammation, prothrombotic condition [33]), leading to more severe disease (large coronary thrombus burden has been noted in most COVID-19 patients suffering STEMI [34]).

COVID-19 infection represents a prothrombotic condition affecting both the venous and arterial system [35, 36, 37, 38, 39]. The etiology of this phenomenon is multifactorial and is possibly related to excessive inflammation/cytokine storm, endothelial injury/dysfunction and subsequent platelet activation [33]. More specifically, during the inflammatory process, various prothrombotic mediators such as von Willebrand Factor (vWF) are released from the dysfunctional endothelial cells that appear to play an important role in the establishment of COVID-19 associated coagulopathy [40]. This observation has potentially important implications in terms of pathogenic link and therapeutic interventions [41], since frequently used drugs in both STEMI and COVID-19 patients (such as statins and heparins) have been shown to regulate the levels and activity of vWF [35, 41, 42].

The first national guidance report on STEMI management was produced by the Chinese Society of Cardiology [18] that accumulated most of the evidence and experience of the first months of the pandemic. This report was the first to highlight fibrinolysis as the initial STEMI treatment in confirmed or suspected COVID-19 patients. PCI would only be recommended in case of fibrinolysis contraindication or failure. This approach aimed to reduce healthcare professionals’ exposure risk, save PPEs and implement a reperfusion treatment timely without delays for COVID-19 status confirmation. Succeeding reports included the expression of various and different opinions [13, 14, 15, 16, 17]. Most reports stressed out the imperative need to test each STEMI patient for COVID-19 as soon as possible after first medical contact. This could help non-COVID-19 patients to be properly treated based on the already established and widely accepted guidelines. SCAI/ACC/ACEP joint report, suggested and enhanced the use of ultra-rapid testing kits when available. Interestingly, the Iranian “247” National Committee report, emphasized the need for PPEs to be applied irrespective of the test result, since diagnostic tools for COVID-19 are not yet validated, and their diagnostic performance remains questionable.

Most guidance documents were produced early after the pandemic outbreak. ESC guidance document has been the most recently updated document but without major differences compared to its early version [13]. Most authors recognized that their documents should not be regarded as strict guidelines as long as they are not completely and adequately evidence-based [17]. Randomized controlled trials comparing fibrinolysis vs PCI as the preferred reperfusion strategy in the era of COVID-19 were not available. Subsequently, expert opinions based on clinical daily practice, practical feasibility and common sense were mainly expressed. Given the significant increase in mortality observed when PCI is not timely delivered, fibrinolysis has been regarded as a potential first step in STEMI therapeutic algorithm since the guideline-recommended time goals were difficult to achieve. Randomized trials to compare fibrinolysis vs PCI have not been conducted so far. Observational data are also scarce and with inconsistent findings, mainly derived from China during the early phase of the pandemic [43, 44, 45]. The implementation of fibrinolysis led to more timely medical interventions [44], but this was not necessarily translated to better clinical outcomes [43]. It appears that a “fibrinolysis-first strategy” has been implemented globally only in a minority of healthcare centers [46, 47], and thus the overall impact of this practice has been probably minor [7, 46]. This could reflect the indisputable role of PCI as the reperfusion method of choice, but also the contribution of the global immunization (either after vaccination or infection) and the development of laboratory methodologies for rapid virus detection [48]. These factors played a very important role and helped the medical community to approach and re-adopt evidence-based practices traditionally implemented before the pandemic [48]. The successful implementation of traditional practices was nicely demonstrated in the study by Ferlini *et al*. [49], where the authors showed that during the second wave of the pandemic in Lombardy, Italy, both COVID-19 and non-COVID-19 STEMI patients received PCI with acceptable time delays.

Another interesting point to consider, is that societies which were in favour of fibrinolysis have been derived mainly from lower income countries with potentially more fragile medical systems [15, 17], or medical systems that faced an enormous COVID-19 burden without time for necessary preparations [18]. International guidelines, on the other hand, were mainly drafted by physicians based on higher income countries with possibly more developed and prepared healthcare systems and adhered to traditional practices in favour of PCI [13, 14]. Guidelines from Taiwan have put fibrinolysis as the first step of reperfusion treatment [15]. In contrast, SCAI/ACC/ACEP recognise primary PCI as the first option in STEMI patients as soon as delays do not exceed a certain time window and comment on strategies adopted in China [14]: *“While fibrinolysis first as a therapeutic strategy has been proposed for COVID-19 STEMI patients based on the experience from Sichuan hospital in China, this might be more applicable in regions with limited primary PCI centers. In the United States, we propose that an initial fibrinolysis therapy be used in non-PCI capable hospitals if the first medical contact to reperfusion is felt to be >120 minutes”.* These differences probably reflect the current daily experience and the amount of resources of each country by the time these recommendations were published.

The management of non-STEMI (NSTEMI) is also briefly discussed in most guidance reports [13, 14, 15, 16, 18]. Most of the researchers agree that in case of very high cardiovascular risk, the STEMI algorithm should be followed. In all other cases, it is highly recommended to confirm NSTEMI diagnosis and to clarify COVID-19 status as soon as possible. NSTEMI diagnosis should be confirmed, as troponin elevation may occur in COVID-19 infection due to various mechanisms which are not fully understood or justified and may not imply atherosclerotic plaque rapture and true acute coronary syndrome (e.g., myocarditis, stress cardiomyopathy, coronary spasm, left ventricular strain, right heart failure, or Type II acute MI due to severe illness) [14, 50]. NSTEMI confirmation is suggested through more specific imaging tests such as coronary computed tomography angiography [13, 14, 15]. COVID-19 status clarification before any intervention is important and most of the times feasible, since NSTEMI usually allows such a justified delay, so that the patient receives optimal treatment according to established guidance protocols. Emphasis is given for both STEMI and NSTEMI patients, always to be carefully assessed in terms of overall clinical condition and prognosis. Patients with critical illness (e.g., severe pneumonia, adult respiratory distress syndrome or intubation due to COVID-19) may not benefit from reperfusion interventions and may be preferably treated with palliative optimal medical treatment [14].

The management of STEMI patients during the pandemic offered some valuable lessons to the medical society. Nearly 3 years after the onset of the pandemic, these lessons can still be useful and meaningful for physicians, especially in low-income countries with low vaccination coverage and/or poor healthcare system organization and during epidemic flares. Personalized and individual patient-tailored treatment decisions should be implemented, especially under unexpected circumstances. Medical staff should be able to optimally implement strategies that are less frequently used but are necessary in certain occasions. Finally, research regarding the feasibility and usefulness of novel strategies such as telemedicine [51], robotic assisted PCI [4] or PCI in prone position [52] will ensure the effective and successful management of patients during future healthcare challenges.

## 5. Conclusions

STEMI management in the era of COVID-19 has been a challenge for healthcare systems and professionals especially during the early and/or peak phases of the pandemic. Global immunization and access to rapid detection of the virus have played an important role so that traditional practices return into daily practice. Future observations will be needed to confirm the true incidence of STEMI in the era of COVID-19 and explain the possibly pandemic-related worse prognosis either in COVID-19 or non-COVID-19 STEMI patients. Most importantly, lessons learned during the COVID-19 pandemic will be a precious legacy for healthcare challenges that may emerge in the future.

## Abbreviations

ACC, American College of Cardiology; ACEP, American College of Emergency Physicians; COVID-19, Coronavirus disease 2019; EAPCI, European Association of Percutaneous Cardiovascular Interventions; ESC, European Society of Cardiology; MI, Myocardial infarction; NSTEMI, Non-ST-segment elevation myocardial infarction; PCI, Percutaneous coronary intervention; PPE, Personal protective equipment; PRISMA, Preferred Reporting Items for Systematic Reviews and Meta-Analyse; SCAI, Society for Cardiovascular Angiography and Interventions; STEMI, ST-segment elevation myocardial infarction; vWF, von Willebrand Factor.

## Supplementary Material

Supplementary material associated with this article can be found, in the online version, at https://doi.org/10.31083/j.rcm2402054.
